# The impact of COVID-19 pandemic on the appointments and anxiety level of Nigerian patients visiting the dental clinics

**DOI:** 10.4314/ahs.v24i2.13

**Published:** 2024-06

**Authors:** Olabimpe Soyoye, Tope Adeyemi, Olayinka Otuyemi, Dada Fadeju, Lillian Enone, Omotoyosi Lawal, Ukachi Nnawuihe, Afolake Salami, Olawale Sotunde

**Affiliations:** 1 University of Medical Sciences, Ondo, Nigeria., Department of Child Dental Health; 2 Bayero University College of Health Sciences, Child Dental Health Aminu Kano Teaching Hospital, Child Dental Health; 3 Obafemi Awolowo University, Child Dental Health; 4 Obafemi Awolowo University, Ile-Ife, Nigeria., Department of Child Dental Health; 5 Lagos State University College of Medicine, Lagos, Nigeria., Department of Restorative Dentistry; 6 University College Hospital, Ibadan, Nigeria, Department of Child Oral Health; 7 University of Medical Sciences, Ondo, Nigeria., Department of Preventive Dentistry; 8 University of Medical Sciences, Ondo, Nigeria, Department of Oral / Maxillofacial Pathology, Radiology and Oral Medicine; 9 Bayero University College of Health Sciences, Department of Restorative Dentistry, Faculty of Dentistry

**Keywords:** COVID-19, dental appointments, anxiety level

## Abstract

**Introduction:**

The epidemic of coronavirus disease 2019 (COVID-19), originating in Wuhan, China, has become a major public health challenge for many countries around the world, including Nigeria. The World Health Organization announced that the outbreaks of the novel coronavirus have constituted a public health emergency of international concern. To control the spread of the disease, many countries, including Nigeria imposed measures such as border screening, social distancing and restriction of all movements. To prevent further spread of the disease, in many places, dentists were restricted to only handling urgencies and emergencies.

**Aim:**

To evaluate the impact of lockdown resulting from COVID-19 pandemic on patients' willingness to attend their dental appointments, clarify their concerns about their ongoing dental treatment, and to assess the anxiety level of patients regarding the risk of contracting the infection at dental offices in Nigeria.

**Method:**

The study was a descriptive cross-sectional study carried out among patients aged 13 years and above, visiting dental clinics in six teaching hospitals across three geopolitical zones of Nigeria: South-West, South-South and North-West.

**Result:**

Few (26.6%) of the participants were compliant with the lockdown restriction. Regarding the general anxiety level of the patients, majority (48.3%) reported calmness. There was a highly statistically significant association between patients' feeling about the pandemic and their willingness to attend a dental appointment visit.

**Conclusion:**

Majority of the participants demonstrated calmness towards the pandemic and did not exhibit fear or anxiety going to dental clinics during the COVID-19 outbreak.

## Introduction

The coronavirus disease (COVID-19) is caused by severe acute respiratory syndrome coronavirus 2 (SARS-CoV-2). It was first reported to the World Health Organisation (WHO) on December 31, 2019.^1^ On January 30, 2020, the WHO declared the COVID-19 outbreak a global health emergency, 2, 3 and on March 11, 2020, it was declared a global pandemic.^4^ The first confirmed case in Nigeria was announced on 27 February 2020, when an Italian citizen in Lagos tested positive for the virus.^5^ Since then, the virus has spread across the different states in the country.

The symptoms of this disease range from mild symptoms like sneezing, running nose, cough, anosmia, ageusia, fever, headache, fatigue, to severe symptoms like difficulty with breathing, persistent chest pain, blue lips or face, confusion and excessive drowsiness, respiratory distress and severe mortality.

The disease mainly spreads between people when they are in close proximity. It spreads very easily and sustainably, primarily via contaminated droplets produced during breathing, coughing, sneezing, talking and singing.

Being highly infective airborne disease, the high infection risk in the dental environment is a serious problem for both dental professionals and patients.^6^ Also, due to the characteristics of dental care, the risk of cross-infection between patients and dental practitioners is high. 7 Pathogenic microorganisms can be transmitted in dental environments by inhaling airborne microorganisms that may remain suspended in the air for long periods, 8 by direct contact with patients' blood or oral fluids, by contact of the conjunctival, nasal or oral mucosae with droplets and aerosols containing microorganisms generated by an infected individual and propelled at close range by coughing and speaking without the use of a mask^9^ and by indirect contact with contaminated instruments and/or environmental surfaces.^10^ Detection rate of SARS-COV-2 in alternative sites and specimens pertaining to dental practice has been extensively reviewed. 11

To control the spread of the disease, many countries, including Nigeria imposed measures such as border screening, social distancing and restriction of all movement, both intra and inter-state movement of people and vehicles, exempting only vehicles carrying food items, fuel, medical supplies and other essential services. Due to the high infective nature of the disease, in many places, dentists were restricted to only handling urgencies and emergencies. Amid the restriction on movement of people and vehicles, dental patients may remain uncertain about whether to attend their dental appointments or not.

A recent study^12^ assessed the impact of quarantine due to the COVID-19 pandemic on dental care in Brazil. The authors reported a significant association between patients' feelings about the COVID-19 pandemic and the level of willingness to attend a dental appointment. Also, the quarantine recommended due to the COVID-19 pandemic was shown to have an impact on dental appointments as majority of the subjects claimed they would visit a dental facility only in case of an emergency. More so, the alarming news on various social media platforms about those infected can generate stress and fear on the general population, particularly those visiting the dental offices.

Dentists need to be familiar with COVID-19 point-of-care (POC) testing options. In addition to contributing to public health, such tests may deliver rapid, accurate, and actionable results to clinical and infection control teams to enhance the safe patient flow in dental practice.^13^ The fear of contracting the COVID-19 disease at the dental office may cause patients' reluctance to seek dental care, and in some cases may lead to delay presentation. A study that evaluated perceived vulnerability to coronavirus infection and the impact on dental practice reported high levels of vulnerability regarding contracting COVID-19 disease, and thus avoiding dental care among the groups of subjects considered.^14^ Delayed dental care has a variety of consequences, whether it is due to reluctance to seek care during a pandemic, dental office closures, or other factors. Routine dental visits are opportunities to provide preventive oral health care (e.g., fluoride treatment and sealants) and to identify oral manifestations of systemic disease that might otherwise be missed.^15^ Delay in presentation for routine dental care thus, can lead to untreated tooth decay and other dental infections.

This study, therefore aimed to evaluate the impact of lockdown resulting from COVID-19 pandemic on patients' willingness to attend their dental appointments, clarify their concerns about their ongoing dental treatment, and to assess the anxiety level of patients regarding the risk of contracting the infection at dental offices in Nigeria.

## Materials and method

The study was a descriptive cross sectional study carried out during the COVID-19 pandemic (between October and November, 2020) among patients aged 13 years and above, visiting dental clinics in six teaching hospitals across three geopolitical zones of Nigeria: South-West, South-South and North-West (University of Medical Sciences Teaching Hospital Complex, Ondo State, Obafemi Awolowo University Teaching Hospital Complex, Osun State, University College Hospital, Oyo State and Lagos State University Teaching Hospital, Lagos State, representing the South-West geopolitical zone; University of Benin Teaching Hospital, Edo State, representing the South-South, and Aminu Kano University Teaching Hospital, Kano State representing the North-West) Ethical approval was obtained from the Ethics and Research committee (ERC) of University of Medical Sciences, Ondo State (Appendix II). Informed consent was obtained from participants that were 18 years and above, while assent was obtained from those below 18 years after informed consent was obtained from their parents/guardians. Inclusion criteria included patients who presented at the dental clinics above, patients already undergoing any form of dental treatment before the pandemic and whose treatment would require multiple appointments and were willing to participate in the study by filling the questionnaire. All patients below 13 years and patients' relatives/guardians were excluded from the study.

The sampling technique was a convenient sampling where well-structured questionnaires were administered to all willing patients who presented at the dental clinics stipulated above at the time this study was conducted. The questionnaire was adapted and modified from questionnaire from a previous study 12 which was an informative survey developed, by which patients could report how they were feeling about the pandemic and how anxious they were about their oral health and ongoing dental treatment. Patients also had support for doubts regarding the questions and answers, if needed. Information sought included socio-demographic characteristics of patients. Other details included questions relating to COVID-19 lockdown and anxiety towards the pandemic. The fear of going to the dentist and cancellation of appointment for fear of infection also were assessed via a dichotomous question (yes/no).

### Statistical analysis

Considering Z crit =1.960, p (pre-study estimate) = 0.95 and Confidence Interval (CI) =0.05, the sample size was calculated to be 292. Data obtained from this study was entered into an excel sheet in a pass worded computer. Data analysis was done using SPSS version 17. Comparison between male and female patients was performed using independent t test. Chi-square, one-way ANOVA and Tukey's tests was also used to evaluate the association of the patients' level of anxiety regarding lockdown/coronavirus pandemic and the willingness to attend dental appointments. Statistical significance was set at P < 0.05.

## Results

Out of the seven hundred questionnaire sent out, four hundred and fifty eight respondents completed the questionnaire. The response rate was 65.4%. The mean age of the participants was 33.98 ± 16.16 years. There were more female patients (56.1%, with a mean age of 32.60 ± 15.47 years) than males (43.9%, mean age 35.76 ±16.86 years). Of the 458 participants, 248(54.2%) were from the South Western part of the country, 176(38.4%) were from the North-West and 34(7.4%) were from the South-South. Others socio-demographic characteristics are as shown in Table 1.

**Table 1:** Socio-demographic Characteristics according to Gender

	Male	Female	Total	p-value
	N= 201 (43.9%)	N= 257 (56.1%)	N= 458 (100%)	
Age (years)				
Mean±SD	35.76 ± 16.86	32.60 ± 15.47	33.98±16.16	
Range	13-85	13-91	13-91	
Occupation				
Schooling	72(15.7%)	116(25.3%)	188(41.0%)	0.006*
Civil servant	56(12.2%)	70(15.3%)	126(27.5%)	
Self employment	59(12.9%)	46(10.0%)	105(22.9%)	
Not working	5(1.1%)	18(3.9%)	23(5.0%)	

Retiree	9(2.0%)	7(1.5%)	16(3.5%)	

* Statistically significant

Majority (91.5%) of the patients had not experienced the COVID-19 infection or its symptoms. (Table 2) With regards to the lockdown compliance, 26.6% of the participants reported that they stayed home during the lockdown period. Majority (60.7%) of the participants reported leaving their homes only when necessary, only 37.9% reported going out as usual, and 4.8% claimed they were not in support of the lockdown as a result of the pandemic. (Table 2)

**Table 2:** Responses to COVID-19 symptoms, lockdown compliance, anxiety to COVID-19, and fear of attending dental clinics, according to gender

	Male	Female	Total	p-value
**Covid symptoms**				
Yes	15(38.5%)	24(61.5%)	39 (8.5%)	0.475
No	186(44.4%)	233(55.6%)	419(91.5%)	
**Lockdown Compliance**				
Didn't leave home	51(41.8%)	71(58.2%)	122(26.6%)	0.937
Left home when necessary	123(44.2%)	155(55.8%)	278(60.7%)	
Going out as usual	17(47.2%)	19(52.8%)	36(7.9%)	
Not in favour of lockdown	10(45.5%)	12(54.5%)	22(4.8%)	
**Anxiety**				
Calm	109(49.3%)	112(50.7%)	221(48.3%)	0.056
Anxious	33(37.5%)	55(62.5%)	88(19.2%)	
Fear	39(44.8%)	48(55.2%)	87(19.0%)	
Indifferent	20(32.3%)	42(67.7%)	62(13.5%)	
**Fear attending dental clinic**				
Yes	47(39.5%)	72(60.5%)	119(26.0%)	0.262

No	154(45.4%)	185(54.6%)	339(74.0%)	

Regarding the general anxiety level of the patients, majority (48.3%) reported calmness, 19.2% claimed they were anxious, 19.0% reported fear of the pandemic while 13.5% were indifferent. Most of the female participants were calm compared to their male counterpacts but this was not statistically significant (p =0.056). Most (74.0%) of the study participants did not experience fear of attending the dental clinic. (Table 2)

Table 3 showed the responses of the participants to COVID-19 symptoms, lockdown compliance and anxiety to COVID-19 based on their locations. COVID -19 symptoms was reported among more of the participants from the northern part of the country. The difference across the groups was highly statistically significant. (p=0.003)

**Table 3:** Responses to COVID-19 symptoms, Lockdown compliance and Anxiety to COVID-19 based on locations

	SW	LOCATION NW	SS	Total	p-value
**COVID symptoms**					
Yes	12(2.6%)	25(5.5%)	2(0.4%)	39(8.5%)	0.003*
No	236(51.5%)	151(33.0%)	32(7.0%)	419(91.5%)	
**Lockdown compliance**					
Didn't leave home	78(17.0%)	32(7.0%)	12(2.6%)	122(26.6%)	0.000*
Left home when necessary	147(32.1%)	110(24.0%)	21(4.6%)	278(60.7%)	
Going out as usual	8(1.7%)	28(6.1%)	0(0.0%)	36(7.9%)	
**Anxiety level**					
Calm	142(31.0%)	59(12.9%)	20(4.4%)	221(48.3%)	0.000*
Anxious	31(6.8%)	56(12.2%)	1(0.2%)	88(19.2%)	
Fear	40(8.7%)	40(8.7%)	7(1.5%)	87(19.0%)	
Indifferent	35(7.6)	21(4.6%)	6(1.3%)	62(13.5%)	
					

* Statistically significant. SW= South-West, NW= North-West, SS= South-South

Participants from the South -West were more compliant with lockdown restriction. A highly statistically significant difference was also seen across the groups. (p= 0.000)

In addition, more of the participants from the South Western part of the country reported calmness to the pandemic compared to others from the other two regions. The difference was highly statistically significant. (p=0.000)

Majority (65.3%) of the participants were undergoing active dental treatment at the time of collection of this data, most of which were females. The types of treatment undertaken were Orthodontic treatment (45.9%), Restorative (31.4%), Endodontic (4.8%), Aesthetic (12.7%), Oral rehabilitation (14.4%) and oral surgical procedures (21.3%).

42% of the total population reported they will go for dental appointment visit, 40.8% would go only in case of emergency, while few, 16.6% would not go for dental appointment during the lockdown period.

Among those under active dental treatment, 46.5% reported they would go for dental appointment during the lockdown, 38.8% would go only in case of emergency, while 14.7% would not go. Most (46.5%) of the participants with ongoing dental treatment would go for dental appointment, while most (44.7%) of the participants not receiving treatment would go only in case of emergency. (Table 4)

**Table 4:** Appointment visit based on whether receiving treatment or not, and appointment cancellation, based on the type of treatment

Appointment visit				
	Yes	Only in case of emergency	No	p value
Dental treatment				
No Treatment	56(35.2%)	71(44.7%)	32(20.1%)	0.055
Treatment	139(46.5%)	116 (38.8%)	44(14.7%)	
**Appointment cancellation**				
	Yes		No	p- value
No treatment	79(49.7%)		80(50.3%)	0.092
Treatment	124(41.5%)		175(58.5%)	
Total	203(100%)		255(100%)	
**Type of treatment**				
Orthodontic	43(41.0%)		62(59.0%)	
Restorative	33(45.8%)		39(54.2%)	
Endodontic	3(27.3%)		8(72.7%)	
Aesthetic	10(34.5%)		19(65.5%)	
Oral rehab	12(36.4%)		21(63.6%)	
Oal surgery	23(46.9%)		26(53.1%)	

Majority of the participants (55.7%) would not cancel their appointment. Most (58.5%) of the patients with ongoing dental treatment reported they would not cancel their appointment for fear of contracting COVID-19. (Table 4)

Regarding concerns reported among respondents about attending a dental appointment, while 27.9% reported risk of getting infected and infecting family members, 17% thought the dental clinic represents a high risk, 15.6% claimed their treatments were not urgent and majority, 39.5% expressed no concernIn addition, most (37.1%) of the participants with ongoing dental treatment expressed no concern attending a dental appointment visit due to COVID-19. (Table 5)

**Table 5:** Concern of attending dental appointment, effect of COVID-19 on treatment, and anxiety to COVID-19 based on whether undergoing active treatment or not

No Ongoing treatment		On-going treatment	Total	p- value
**Concern of appointment visit**				
Risk of getting infected	44(27.7%)	84(28.1%)	128(27.9%)	0.251
Dental clinic is a high risk	20(12.6%)	58(19.4%)	78(17.0%)	
My treatment not urgent	25(15.7%)	46(15.4%)	71(15.5%)	
No concern	70(44.0%)	111(37.1%)	181(39.5%)	
**Concern of effect of Covid on ongoing treatment**				
Delay treatment	53(33.3%)	150(50.2%)	203(44.3%)	0.000*
Lose investment	26(16.4%)	52(17.4%)	78(17.0%)	
Not worried	80(50.3%)	97(32.4%)	177(38.6%)	
**Anxiety level**				
Calm	75(47.2%)	146(48.8%)	221(48.3%)	0.029*
Anxious	23(14.5%)	65(21.7%)	88(19.2%)	
Fear	41(25.8%)	46(15.4%)	87(19.0%)	
Indifferent	20(12.6%)	42(14.0%)	62(13.5%)	

* Statistically significant

Considering the concern expressed by participants about how the lockdown could affect their on-going dental treatment, while 44.3% were concerned about a delay, as they were anxious to finish treatment, 17.0% were worried about losing the time and money they have invested in the treatment or the situation of their mouth/teeth getting worse, and 38.6% of the participants were not worried. Most of the participants with ongoing dental treatment were more worried about a delay in their treatment. There is a statistically significant difference between those receiving treatment and those not receiving treatment showing that those receiving treatment were more worried about the effect the pandemic could have on their ongoing treatment, while most of those not receiving treatment were not worried. (Table 5)

Regarding the general anxiety level to the pandemic, there is a statistically significant difference between those undergoing treatment and those not, showing that those undergoing active treatment are calmer than those not receiving treatment. (Table 5)

Comparing the responses amongst the different treatment procedures being received, most of those receiving orthodontic treatment reported to be calmer compared to those receiving other forms of dental treatment. A high statistical significant difference existed across the groups. p = 0.000 (Table 6)

**Table 6:** Anxiety to COVID-19 and effect of COVID-19 on ongoing treatment, based on type of treatment

Anxiety level					
	Calm	Anxious	Fear	Indifferent	p-value
Orthodontics	50(34.2%)	19(29.2%)	17(37.0%)	19(45.2%)	0.000*
Restorative	40(27.4%)	10(15.4%)	8(17.4%)	14(33.3%)	
Endodontic	3(2.1%)	5(7.7%)	3(6.5%)	0(0.0%)	
Aesthetic	6(4.1%)	19(29.2%)	3(6.5%)	1(2.4%)	
Oral rehab	20(13.7%)	5(7.7%)	5(10.9%)	3(7.1%)	
Oral Surgery	27(18.5%)	7(10.8%)	10(21.7%)	5(11.9%)	

Concern of effect of covid-19 on ongoing treatment					
	Delay treatment		Lose investment	Not worried	p-value
Treatment Orthodontics	62(59.0%)		15(14.3%)	28(26.7%)	0.004*
Restorative	35(48.6%)		12(16.7%)	25(34.7%)	
Endodontics	5(45.5%)		2(18.2%)	4(36.4%)	
Aesthetic	9(31.0%)		6(20.7%)	14(48.3%)	
Oral rehabilitation	20(60.6%)		4(12.1%)	9(27.3%)	
Oral Surgery	19(38.8%)		13(26.5%)	17(34.7%)	

Among the participants with ongoing dental treatment, most (59.0%) of those undergoing orthodontic treatment were more concerned about a delay in their treatment than those receiving treatment from other dental specialties. (Table 6)

Comparing the responses among genders, while most of the males(40.3%) reported they would go for their appointment visit only in case of emergency, most of the females(117,45.5%) reported they would go for their appointment visit if the dentist schedule an appointment, regardless of the pandemic. However, no statistically significant difference existed among the genders. In addition most of the females were more worried about a delay in their on-going dental treatment as a result of the pandemic than the males. (Table 7)

**Table 7:** Response to appointment visits, and effect of COVID-19 pandemic on dental treatment according to gender

	M	F	p-value
**Appointment visit**			
Yes	78(38.8%)	117(45.5%)	0.074
Only in emergency	81(40.3%)	106(41.2%)	
No	42(20.9%)	34(13.2%)	
**Effect of Covid-19 on treatment**			
Delay treatment	83(41.3%)	120(46.7%)	0.498
Lose investment	37(18.4%)	41(16.0%)	
Not worried	81(40.3%)	96(37.4%)	

There was a highly statistically significant association between patients' feeling about the pandemic and their willingness to attend a dental appointment visit. Patients who were calm were more willing to attend a dental appointment than those anxious, afraid or indifferent. More of the patients who reported anxiety and fear would attend a dental appointment only in case of emergency. (Table 8) Regarding precautionary measures to avoid contamination during the pandemic, majority(239, 52.2%) of the participants suggested all the precautionary measures, that is, disposable lab coat and N95 surgical mask for dentists (which should be changed in between patients), avoiding close contact with other patients in the waiting room, personal protective equipments for patients (in form of nose masks, foot wear and disposable apron) and alcohol gel at reception for patient use.

## Discussion

The outbreak of COVID-19 pandemic engendered much uncertainty and risks, and disrupted normal life across the globe with Nigeria not being an exception. Nigeria reported her first case of the disease in February 2020, and following global trend, restrictive measures including a lockdown were introduced by the government for more than four months. This significantly negatively impacted not only the economy, education and social relations but also health services including dental care delivery. Following WHO recommendations, elective dental procedures including Orthodontic treatment and other forms of dental treatment were put on hold, therefore dental treatments were restricted to conduct only on emergencies situations.^16^

The results of this study showed that majority of the participants were compliant with, and respected the lockdown restriction declared by the federal government as most of the patients were going out only when necessary, such as to buy foodstuff or medicine, and many didn't leave their homes, all through the period of restriction. This high level of compliance probably may be due to information and misinformation from media about such factors as those associated with the mode of transmission of the virus, incubation period, the impact on the socio-economic, political and psychological livelihood of people as well as the fatality rate of the disease in other countries of the world, therefore the need to avoid unnecessary movements. In addition, recent studies affirm the high and huge level of psychosocial consequences of outbreaks like COVID-19 on individuals, the general public, and the international community.^17^, ^18^

Although there is an increased level of awareness of the seriousness of the pandemic, many of the participants of this study reported calmness towards the pandemic compared to few who reported anxiety and fear towards the situation. This finding is consistent with findings of a recent study in Nigeria which revealed that majority of the residents in Nigeria insignificantly had moderate anxiety symptoms during the COVID-19 pandemic.^19^ This finding may be due to reduced number of cases and fatality rate reported in the country compared to high number recorded in other countries of the world.^20^

However, despite the awareness on media of the risk of contracting the disease during dental procedures, majority of the participants of this study reported not having fear going to the dental clinic during the corona virus pandemic. This may be linked to the level of calmness reported towards the pandemic by the participants.

The focus of this study was to assess the effect of COVID-19 pandemic on dental appointments and to evaluate the anxiety levels for COVID-19 on patients visiting the dental clinics. Of all the participants of this study, majority reported they will go for dental appointment visit, many reported they would go only in case of emergency, while few reported they would not go if the dentist called to schedule an appointment during the lockdown period.

In addition, the results of this study showed that majority of the participants with ongoing dental treatment would not cancel their appointments for fear of contracting COVID-19. This willingness to attend dental appointments despite the risk of contracting the infection may probably be because patients in the middle of a course of treatment may show greater concern and care towards their treatment, and may not readily miss an appointment visit because of the negative impact it may have on the outcome of their treatment.

Furthermore, considering the concern expressed by participants, of attending a dental appointment during the pandemic, most of those undergoing dental treatment expressed no concern. This finding may be because the participants undergoing active dental treatment are rather more concerned of the effect that the pandemic may have on their ongoing treatment than the situation of the COVID-I9 pandemic outbreak itself. This is clearly evidenced in the results of this study which showed that most of the patients with ongoing dental treatment expressed more concern on the effect the pandemic may have on their ongoing dental treatment as they were mostly worried about the delay they might experience in finishing their treatment as a result of the pandemic and also the concern of losing the time and money they have invested in the treatment. This may also be the reason why majority of these participants, that is, those undergoing active treatment reported to be calm considering their feelings to the pandemic situation and are also willing to attend their appointment visits if the dentist called to schedule an appointment.

The findings of this study also showed that most orthodontic patients reported to be calm regarding their feelings to the pandemic outbreak, and were more worried about a delay in finishing their treatment compared to other patients receiving treatment from other dental specialties. This concern expressed by orthodontic patient is justifiable since the duration of orthodontic treatments is usually longer than most other dental treatments.^21^ In addition, each missed orthodontic appointment has been shown to increase the treatment time by about one month. 22, 23

Comparing the findings of this study among sexes, the females were more compliant with the lockdown restrictions as most of them left their homes only when necessary or didn't leave at all. Also, most of the females reported they were calm regarding their feelings to the COVID-19 pandemic, and were more willing to attend dental appointments during the pandemic compared to the males, most of who reported they will attend dental appointments only in case of emergency. This finding contradicts the report of a previous study that showed that women are more emotionally affected by difficult situations and are more likely than men to show symptoms of anxiety and fear.^24^ However, the finding is consistent with previous study that showed that females are more compliant with dental treatment than men 25 and the general anxiety level to the pandemic did not affect their willingness to attend dental appointments. Furthermore, most of the females in the studies were worried about a delay in finishing their treatment than males. This eagerness to finish their treatment on time may be what reinforced their willingness to attend dental appointments during the pandemic. In addition, females have been shown to be more concerned about their aesthetics than men 26,27 However, these findings are contrary to the findings of similar study by Peloso et al 12 who reported that the women were less worried abut a delay in reaching the end of treatment than men, shared they were more anxious and afraid of the COVID-19 pandemic than men, and their feelings of anxiety and fear in that study affected their willingness to attend a dental appointment.

Comparing the findings among the different locations in Nigeria, COVID-19 symptoms were reported among more of the participants from the North. This probably may be due to the fact that at the time of this survey, the North was the epicentre of the pandemic in the country. In addition, despite the fact that most of the participants were compliant with the restriction on movement during the pandemic, a higher percentage of those who were not compliant were from the North. This may also account for the higher number of self- reported COVID-19 cases recorded in the North in this study. This may also be the reason why a higher percentage of the respondents who reported anxiety and fear towards the pandemic were from the North.

The results of this study showed that regardless of gender or locations, there was a strong association between feelings about the pandemic and the willingness to attend dental appointment. Majority of those who are calm and indifferent were more willing to attend dental appointments during the pandemic, while most of those who reported anxiety and fear would attend an appointment only in case of emergency. This finding may be linked to the fact most of those who reported calmness are those with ongoing dental treatment, and therefore would not entertain interferences with their appointment visits, that may prolong their treatment time.

Preventing the spread of SARS-CoV-2 requires new management strategies that may differ from those used to manage other diseases.^28^,^29^ Hence various treatment guidelines have been developed by the Centers for Disease Control and Prevention (CDC),^30^ the American Dental Association (ADA), 31 World Health Organisation (WHO)^32^ and others to prevent spread of the infection. The findings of this study showed that the participants, whether undergoing active dental treatment or not, are aware of these guidelines, as majority of them considered all the precautionary measures important in the prevention of transmission of COVID-19 in the dental clinic.

## Conclusion

1. The COVID-19 pandemic did not have a perceived negative influence on dental appointment visits as majority of the participants, particularly those with ongoing dental treatment were willing to attend dental appointment regardless of the risk of doing so.
2. Majority of the participants demonstrated calmness towards the pandemic and did not exhibit fear or anxiety going to dental clinics during the COVID-19 outbreak.
